# Tim-3 Expression Defines Regulatory T Cells in Human Tumors

**DOI:** 10.1371/journal.pone.0058006

**Published:** 2013-03-05

**Authors:** Jing Yan, Yi Zhang, Jing-Ping Zhang, Jing Liang, Lian Li, Limin Zheng

**Affiliations:** 1 State Key Laboratory of Oncology in South China, Sun Yat-sen University Cancer Center, Guangzhou, P. R. China; 2 State Key Laboratory of Biocontrol, School of Life Sciences, Sun Yat-sen University, Guangzhou, P. R. China; Centre de Recherche Public de la Santé (CRP-Santé), Luxembourg

## Abstract

Tim-3, a member of the novel Tim (T cell immunoglobulin and mucin domain) family, has been reported to negatively regulate the immune responses against viral infection and had implications for autoimmune disease. However, the nature and role of Tim-3^+^ CD4 T cells in human tumors remain largely unknown. In the present study, we characterized Tim-3^+^ CD4 T cells in 100 specimens from human hepatocellular, cervical, colorectal and ovarian carcinoma patients. Compared with peripheral blood and nontumor-infiltrating lymphocytes, the lymphocytes isolated from the corresponding tumor tissues of hepatocellular, cervical, colorectal and ovarian carcinoma patients contained significantly greater proportion of Tim-3^+^ CD4 T cells. The majority of tumor-derived Tim-3^+^ CD4 T cells exhibited an impaired capacity to produce IFN-γ and IL-2, but expressed higher levels of CD25, Foxp3, CTLA-4 and GITR than their Tim-3^−^ CD4 T cell counterparts. In contrast, most Tim-3^+^ CD4 T cells isolated from the paired nontumor tissues and peripheral blood did not express these molecules. Moreover, tumor-derived Tim-3^+^ CD4 T cells, but not tumor-derived Tim-3^−^ CD4 T cells, significantly suppressed the proliferation of autologous CD8^+^ T cells *in vitro*. Notably, multi-color immunofluorescence and confocal microscopy demonstrated that Tim-3^+^Foxp3^+^CD4^+^ cells were preferentially distributed in the tumor nest rather than the peritumoral stroma of hepatocellular carcinoma. Together, our data indicate that Tim-3-expressing CD4 T cells in human tumors could represent the functional regulatory T cells which contribute to the formation of the immune-suppressive tumor micromilieu.

## Introduction

Tim-3, a member of the novel T cell immunoglobulin and mucin domain (Tim) family, was originally identified as a negative immune regulator which is expressed on T helper 1 (Th1) cells, but not Th2 cells [1]. The interaction between Tim-3 and its ligand galectin-9 inhibits Th1 responses and induces peripheral tolerance [2]–[4]. However, growing evidence indicates that Tim-3 is also expressed on multiple cell types [5], [6] and is involved in the pathogenesis of autoimmune disease and virus infection [7]. For example, T cell clones isolated from multiple sclerosis patients expressed lower levels of Tim-3 but secreted higher amounts of IFN-γ [8]. Upregulation of Tim-3 expression has been observed on exhausted CD8 T cells isolated from patients infected with human immunodeficiency virus (HIV), Hepatitis B virus (HBV), Hepatitis C virus (HCV) and Herpes simplex virus (HSV), and correlated with inefficient antiviral activity [9]–[13]. Recently, Tim-3 expression on Foxp3^+^ Tregs was found in mouse skin transplantation model and chronic HCV-infected patients [14], [15]. Such dysregulation of Tim-3 expression in disease settings indicates that Tim-3 may play an important role in adaptive immune responses.

Increased expression of Tim-3 has been recently detected in tumor-infiltrating lymphocytes (TILs) [16]–[20]. In murine tumor models, coexpression of Tim-3 and Programmed death 1 (PD-1) in CD8 T cells marks the most exhausted population of tumor-infiltrating T cells [17]. Simultaneous blockade of Tim-3 and PD-1 could effectively restore the antitumor activity of these exhausted T cells, resulted in complete tumor regression, and remained tumor free even after rechallenge [17]. In addition, expression of Tim-3 on T cells has been shown to promote the expansion of immunosuppressive myeloid-derived suppressor cells in mice bearing T-cell lymphomas [21], suggesting that Tim-3 is an inhibitory regulatory molecule important for the induction of immune tolerance in tumors microenvironment. Nonetheless, other studies have indicated that Tim-3 also plays a positive role in the antitumor immune response [22]–[24]. The Tim-3 ligand galectin-9 was found to prolong the survival of tumor-bearing mice by inducing cytotoxicity in CD8^+^Tim-3^+^ T cells, as well as facilitating the maturation of Tim-3^+^ dendritic cells [24]. Although these findings suggest that Tim-3^+^ cells have a potential impact in murine tumors, the nature and role of Tim-3^+^ CD4 T cells in human tumors are still largely unknown [7].

In the current study, we report that Tim-3^+^ CD4 T cells accumulate in human tumor tissues, and that Tim-3 expression defines a subset of regulatory T cells (Tregs) in several types of human tumors. Tim-3^+^ Tregs from human hepatocellular carcinoma (HCC) suppressed autologous CD8 T cell proliferation and cytokine production *in vitro*. Additionally, Tim-3^+^ Tregs specifically accumulated in the tumor nest, where Tim-3^+^ CD4 cells had close contact with the Tim-3 ligand galectin-9. Together, our results suggest that the Tim-3-galectin-9 pathway may contribute to the suppressive tumor microenvironment in human cancer by promoting regulatory T cells.

## Materials and Methods

### Patients and Specimens

A total of 90 patients with pathologically confirmed hepatocellular carcinoma and 10 patients with cervical, colorectal or ovarian carcinoma from Sun Yat-sen University Cancer Center were enrolled in this study. None of the patients had received antitumor therapy before sampling. Paired tumor and nontumor tissue samples from 46 HCC patients and 10 patients with cervical, colorectal or ovarian carcinoma were processed to isolate fresh tissue infiltrating leukocytes. Fresh blood samples from 31 HCC patients were processed to isolate peripheral blood mononuclear cells (PBMCs). Paraffin-embedded samples from 13 HCC patients were used for immunofluorescence staining. Control blood samples were obtained from 36 healthy donors attending Guangzhou Blood Center, all of whom were negative for HBV, HCV, HIV and syphilis. The clinical and pathological characteristics of the patients are summarized in Table 1 and Tables S1, S2, S3.

**Table 1:** Clinical and pathological characteristics of the HCC patients.

Variable	Fresh tissue	Peripheral blood	Paraffin section
Cases (*n*)	46	31	13
Age, years (median, range)	51, 22–79	59, 22–76	59, 36–80
Gender (male/female)	43/3	25/6	13/0
HBsAg (negative/positive)	3/43	7/24	3/10
Cirrhosis (absent/present)	10/36	5/26	3/10
ALT, units/L (median, range)	38, 14–303	37, 16–69	60, 29–82
AFP, ng/ml (≤25/>25)	13/33	10/21	4/9
Tumor multiplicity (solitary/multiple)	32/14	22/9	9/4
Tumor size, cm (≤5/>5)	11/35	17/14	2/11
Vascular invasion (absent/present)	38/8	25/6	12/1
TNM stage (I–II/III–IV)	25/21	19/12	8/5
Tumor differentiation (I–II/III–IV)	19/27	14/17	6/7

Note: HBsAg, hepatitis B surface antigen; ALT, alanine aminotransferase; AFP, a-fetoprotein; TNM, tumor-node-metastasis.

### Ethics Statement

Written informed consent was obtained from all patients and all samples were coded anonymously, in strict accordance with local ethical guidelines and as stipulated by the Declaration of Helsinki. Before the study, the protocol was approved by the Review Board of Sun Yat-sen University Cancer Center.

### Cell Isolation

Peripheral blood leukocytes were isolated by Ficoll density gradient centrifugation. Fresh tumor and nontumor tissues were washed, cut into small pieces and digested in RPMI 1640 supplemented with 0.05% collagenase IV (Sigma-Aldrich, St. Louis, MO), 0.002% DNase I (Roche, Basel, Switzerland), and 20% FBS (HyClone Laboratories, Logan, UT) at 37°C for 30 minutes. Dissociated cells were filtered through a 150-µm mesh. Tumor-infiltrating lymphocytes (TILs) and nontumor-infiltrating lymphocytes (NILs) were obtained after Ficoll density gradient centrifugation [25]–[28].

### Flow Cytometric Analysis

The fluorochrome-conjugated antibodies (Abs) and isotype-matched controls used in this study are described in Table S4. The non-specific staining for Tim-3 expression was shown in Figure S1. For intracellular cytokine staining, the cells were stimulated for 4 h with Leukocyte Activation Cocktail (BD Biosciences, San Diego, CA) and then stained with extracellular Abs, fixed and permeabilized with IntraPre Reagent (Beckman Coulter, Fullerton, CA), and finally stained with intracellular Abs [25]. To be noticed, stimulation with Leukocyte Activation Cocktail for 4 h did not affect Tim-3 expression on T cell surface. Intracellular staining for Foxp3 was performed by using a Foxp3 staining kit (eBioscience, San Diego, CA) according to the manufacturer’s instructions. Data were acquired using a Gallios multicolor flow cytometer (Beckman Coulter, Brea, CA) and analyzed with FlowJo software (TreeStar, San Carlos, CA).

### Immunofluorescence

Paraffin-embedded samples were cut into 5-µm sections and processed for immunofluorescence staining, as previously described [27]. Sections were dewaxed in xylene and rehydrated in a series of graded alcohols according to histopathological standards. Antigen retrieval was achieved by pressure cooking in EDTA antigen retrieval buffer (pH 9.5, Zhongshan golden bridge, China) for 10 min and cooling them to room temperature. Slides were then incubated in PBS containing 5% BSA and 0.1% Triton X-100 for 30 minutes to block unspecific binding. HCC tissue sections were stained with rabbit-anti-human CD4 (Spring Bioscience, Fremont, CA) or mouse-anti-human CD4 (NeoMarker, Freemont, CA), goat-anti-human Tim-3 (R&D Systems, Minneapolis, MN), mouse anti-human Foxp3 (Abcam, Cambridge, UK), mouse anti-human CD68 (Dako, Glostrup, Denmark) and rabbit anti-human galectin-9 (Abcam), followed by Alexa Fluor 488-, 555-, or 647-conjugated donkey anti-mouse IgG, Alexa Fluor 555- or 647-conjugated donkey anti-rabbit IgG, or Alexa Fluor 488-conjugated donkey anti-goat IgG, as appropriate (Molecular Probes, Carlsbad, CA). Negative control staining was performed by replacing the primary antibodies with isotype-matched control antibodies. Nuclei were stained with 40-6-diamidino-2-phenylindole (DAPI). Images were viewed and assessed using an Olympus confocal laser microscope (Fluoview; Tokyo, Japan). For the quantification of CD4^+^Tim-3^+/−^Foxp3^+/−^ subset cells, CD4^+/−^galectin-9^+/−^ subset cells, or CD68^+/−^galectin-9^+/−^ subset cells, slides were first screened for CD4 or CD68 hot-spots. Then 8 images per slide were taken with 40× oil-immersed objective lens. Numbers of each subset cell per field were counted manually by two independent, blinded observers.

### Suppression Assay

The suppression assay was performed as previously described with minor modifications [29]. TILs were first gated on CD45^+^ cells to exclude non-hematopoietic CD45^–^ cells. CD4^+^Tim-3^+^, CD4^+^Tim-3^−^ and CD4^−^ T cells were sorted using a FACS Aria II (BD Biosciences, San Diego, CA); cell purity was over 90%. For suppression assay using carboxyfluorescein diacetate succinimidyl ester (CFSE; Molecular Probes), 5 × 10^4^ CD4^−^ T cells (responder) were labeled with 1 µM CFSE and cultured with autologous CD4^+^Tim-3^+^ cells, CD4^+^Tim-3^−^ T cells or medium alone. Cells were first stimulated with anti-CD3 (0.2 µg/ml) and anti-CD28 (0.4 µg/ml) mAbs for 2 days, cultured in the presence of IL-2 (20 U/ml) for another 3 days, and then cell proliferation was analyzed by flow cytometry. For the BrdU incorporation assay, the cells were cultured as stated above and then pulsed with BrdU during the last 6 h using the BrdU kit (Roche, Mannheim, Germany). Cell proliferation was quantified by measuring the optical density (OD) of each group. Culture supernatants were collected for quantification of the IFN-γ concentration by ELISA (eBioscience, San Diego, CA).

### Statistical Analysis

Data are presented as the mean ± SEM. Comparisons between groups were performed using the Student’s *t*-test. The association between indexes of Tim-3^+^ Tregs and the TNM stages were evaluated using Fisher’s exact test. Statistical analyses were performed using SPSS software (version 13.0; SPSS Inc., Chicago, IL).

## Results

### Tim-3 Expression is Upregulated on CD4 T cells in the Tumor Tissues of Cancer Patients

**Figure 1:**
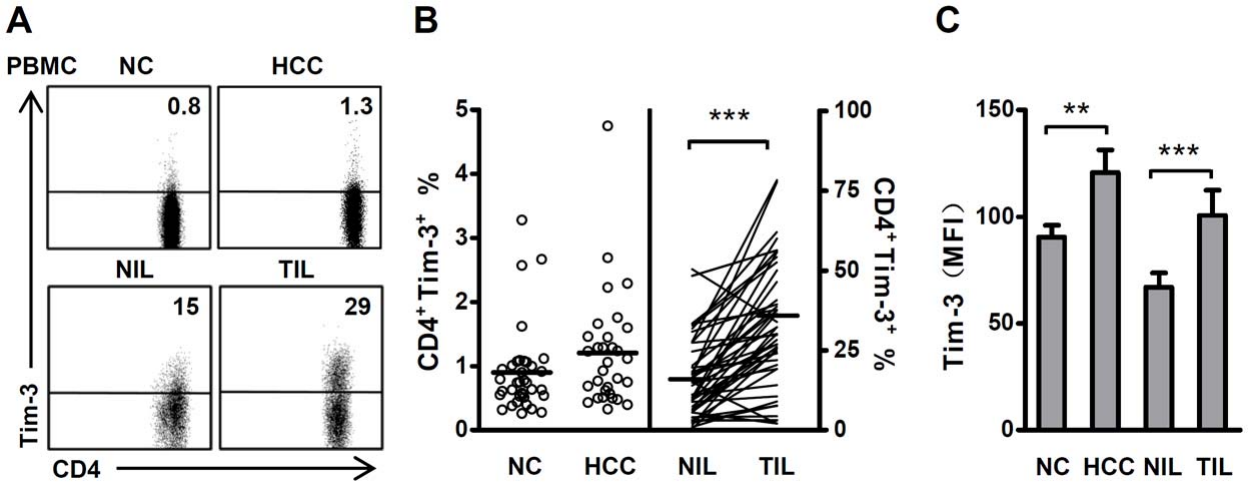
Tim-3 is upregulated on CD4 T cells in HCC tumor tissues. A . Representative FACS analysis of Tim-3 expression in CD4 T cells isolated from the PBMCs of normal healthy controls (NC) and HCC patients (HCC), and nontumor-infiltrating lymphocytes (NILs) and tumor-infiltrating lymphocytes (TILs) from HCC patients. Flow cytometry data are representative of at least 31 samples in each group. **B**. Statistical analysis of the proportion of Tim-3^+^ CD4 T cells in NC (*n* = 36), HCC (*n* = 31), NILs and TILs (*n* = 46). Each circle or thin line represents a single case. Horizontal lines indicate the mean of an individual group. **C**. Mean fluorescence intensity (MFI) for Tim-3 on CD4^+^Tim-3^+^ T cells in each group. NC (*n* = 36), HCC (*n* = 31), NILs and TILs (*n* = 46). Bars indicate the SEM; **, *P*<0.01; ***, *P*<0.001.

To explore the potential role of Tim-3 in tumor immunopathology, we first examined the distribution of Tim-3 in lymphocytes freshly isolated from the paired tumor and nontumor tissues of 46 HCC patients, as well as lymphocytes isolated from the peripheral blood of 31 HCC patients and 36 healthy donors. Approximately 5% of the circulating Tim-3^+^ lymphocytes in both healthy donors and HCC patients were CD4 T cells. This proportion was significantly elevated in tumor-infiltrating lymphocytes (TIL) compared with nontumor-infiltrating lymphocytes (NIL) (19.9±2.7% vs. 5.3±0.9%, *P*<0.001, Figure S2). With respect to CD4 T cells, Tim-3 was only expressed on a small fraction of the circulating CD4 T cells in healthy individuals, with slightly higher levels of circulating Tim-3^+^ CD4 T cells observed in HCC patients (Figure 1A and 1B). In contrast, we detected substantial numbers of Tim-3-expressing CD4 T cells in both nontumor and tumor tissues. The frequency of Tim-3^+^ CD4 T cells and mean fluorescence intensity (MFI) for Tim-3 were significantly higher in TILs than the NILs of the same HCC patients (32.9±2.8% vs. 16.5±1.9% for frequency; 100.7±11.7 vs. 67.1±6.7 for MFI, respectively; both *P*<0.001, Figure 1B and 1C).

To investigate whether these findings are confined to HCC, we also examined Tim-3-expressing T cells in other human tumors. Compared with the corresponding nontumor tissues, the levels of Tim-3-expressing CD4 T cells were also significantly higher in tumors from patients with colorectal, cervical or ovarian carcinoma (Figure S3A). These data indicated that Tim-3-expressing cells comprise a significant subset of CD4 T helper cells, which are selectively enriched in human tumors.

### Tumor-derived Tim-3^+^ CD4 T cells Exhibit Impaired Production of IFN-γ and IL-2

**Figure 2:**
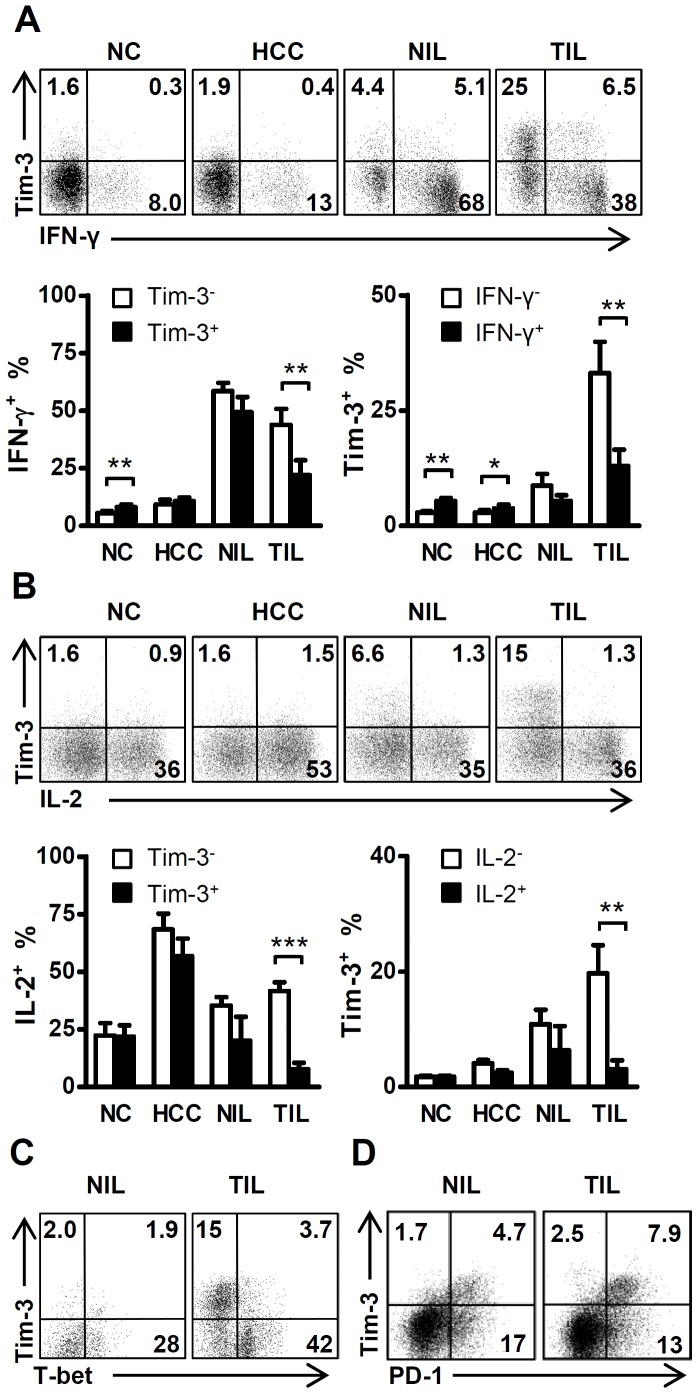
Tumor-infiltrating Tim-3^+^ CD4 T cells exhibit impaired ability to produce Th1-type cytokines. A . Representative FACS analysis illustrating the association between IFN-γ and Tim-3 expression in CD4 T cells isolated from the PBMCs of normal healthy controls (NC, *n* = 15) and HCC patients (HCC, *n* = 10), and nontumor-infiltrating lymphocytes (NILs, *n* = 6) and tumor-infiltrating lymphocytes (TILs, *n* = 6) from HCC patients. Statistical analysis showed the mean percentage of IFN-γ^+^ cells in the Tim-3 positive or negative CD4 T cell subsets (left), and the proportion of Tim-3^+^ cells in the IFN-γ positive or negative CD4 T cell subsets (right). **B**. Relationship between the expression of Tim-3 and IL-2 in CD4 T cells, At least 4 samples were tested in each group. Bars indicate the SEM; *, *P*<0.05; **, *P*<0.01; ***, *P*<0.001. **C**. The majority of Tim-3^+^ CD4 T cells isolated from TILs did not express the Th1-specific transcription factor T-bet, *n = *4. **D**. Correlation of Tim-3 and PD-1 expression in tumor tissues, *n = *4.

CD4 T helper (Th) cells play a central role in orchestrating host immune responses through cytokine production and expression of membrane-bound molecules [30]–[32]. To evaluate the functional status of Tim-3^+^ CD4 T cells, we examined their capacity to produce IFN-γ, IL-2, IL-4 and IL-17. In TILs isolated from HCC tissues, Tim-3^+^ CD4 T cells produced significantly less IFN-γ and IL-2 than the Tim-3^−^ CD4 T cells (22.1±6.4% vs. 43.9±6.9%, *P*<0.01 for IFN-γ; 7.8±2.8% vs. 41.7±3.7%, *P*<0.001 for IL-2, respectively; Figure 2A and 2B). The frequency of Tim-3^+^ CD4 T cells from TILs was also significantly lower in IFN-γ^+^ or IL-2^+^ cells than their IFN-γ^−^ or IL-2^−^ counterparts (13.0±3.5% vs. 33.1±6.8% for IFN-γ; 3.1±1.5% vs. 19.7±4.9% for IL-2, both *P*<0.01, Figure 2A and 2B). In contrast, we found little or no difference in IL-2 or IFN-γ production between Tim-3^+^ and Tim-3^−^ CD4 T cells derived from NILs, whereas circulating Tim-3^+^ CD4 T cells produced even more IFN-γ than Tim-3^−^ cells (Figure 2A), suggesting that the tumor environment can profoundly affect the phenotype of Tim-3^+^ CD4 T cells. Sorted Tim-3^+^ CD4 T cells also secreted less amounts of IFN-γ and IL-2 compared with Tim-3^−^ CD4 T cells (data not shown). In addition, most Tim-3^+^ CD4 T cells did not produce IL-4 or IL-17 (Figure S4). Similar results were observed in samples isolated from human patients with colorectal, cervical and ovarian carcinoma, including the selective impairment of IFN-γ and IL-2 production in Tim-3^+^ CD4 T cells isolated from TILs (data not shown). These data provide the first indication that Tim-3-expressing CD4 T cells might represent a population of dysfunctional Th1 cells (or another cell type) in human tumor tissues.

To determine whether tumor-derived Tim-3^+^ CD4 T cells are dysfunctional Th1 cells, we quantified expression of the Th1-specific transcription factor T-bet [33] and the well-recognized exhausted/anergic marker PD-1 [34], [35] on Tim-3^+^ CD4 T cells. The majority of Tim-3^+^ CD4 T cells isolated from TILs did not express T-bet (Figure 2C), suggesting that the majority of these cells were different to Th1 cells. In contrast, approximately 80% of the Tim-3^+^ CD4 T cells isolated from TILs co-expressed PD-1 (Figure 2D), a marker for exhausted or anergic T cells.

### Tim-3 is Preferentially Expressed by CD25^+^CD127^low^Foxp3^+^ T cells in Human Tumors

**Figure 3:**
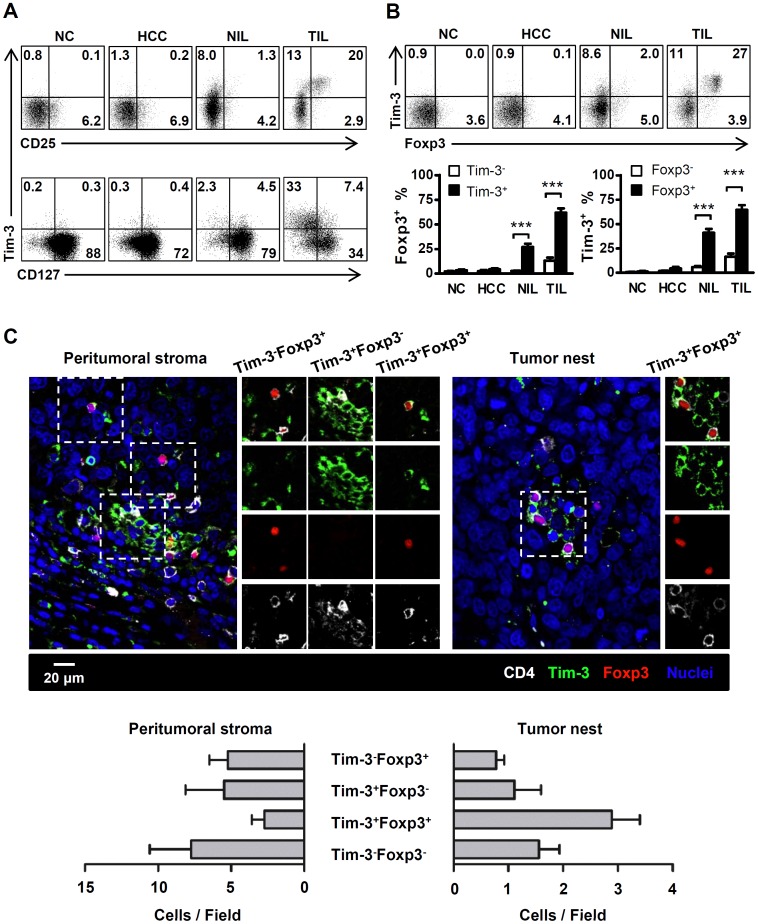
Tim-3 expression defines a population of CD4 T cells with Treg characteristics in TILs. A . Representative FACS analysis showing the association of CD25, CD127 and Tim-3 expression in CD4 T cells isolated from the PBMCs of normal healthy controls (NC, *n* = 7) and HCC patients (HCC, *n* = 6), and nontumor-infiltrating lymphocytes (NILs, *n* = 18) and tumor-infiltrating lymphocytes (TILs, *n* = 18) from HCC patients. **B**. FACS and statistical data showing that most tumor-infiltrating Tim-3^+^ CD4 T cells expressed the Treg-specific transcription factor Foxp3. Bars indicate the SEM; ***, *P*<0.001. **C**. Paraffin-embedded HCC sections were subjected to multi-color immunofluorescence staining for CD4 (white), Tim-3 (green), Foxp3 (red) and nuclei (DAPI, blue). Confocal microscopic analysis revealing the different distribution of Tim-3^+^Foxp3^+^CD4^+^ cells in the peritumoral stroma and tumor nest of HCC. Images are representative of 13 samples. For the quantification of CD4^+^Tim-3^+/−^Foxp3^+/−^ cells, eight CD4 hot-spot fields per slide were taken with 40× oil-immersed objective lens. Numbers of each subset cells were counted manually by two independent, blinded observers.

To further characterize the phenotype of Tim-3^+^ CD4 T cells, we examined the expression of CD25 and CD127. As shown in Figure 3A, Tim-3^+^ CD4 T cells isolated from TILs expressed significantly higher levels of CD25 than the Tim-3^−^ cells isolated from TILs (63.2±5.0% vs. 13.1±2.1%, *P*<0.001, Figure S5A). Interestingly, the majority (82.9±4.7%) of Tim-3^+^ CD4 T cells in TILs were CD127^low^ (Figure S5B). The frequency of Tim-3^+^ CD4 T cells was also significantly higher among CD25^+^ or CD127^low^ cells than their CD25^−^ or CD127^high^ counterparts (Figure 3A and S5); these characteristics are also shared by human Treg cells [30], [36]-[38]. Thus, we next determined the correlation between expression of Tim-3 and Foxp3, a transcription factor generally used as a marker for Treg cells [39]. As shown in Figure 3B, Foxp3 expression was significantly higher in Tim-3^+^ CD4 T cells from TILs than the Tim-3^−^ cells (61.7±4.5% vs. 13.1±3.2%, *P*<0.001), at a level similar to CD4^+^CD25^+^ T cells (65.5±4.7%). Back gating of Foxp3 in TILs showed that ∼70% of the CD4^+^Foxp3^+^ T cells were Tim-3^+^, compared with ∼20% of the CD4^+^Foxp3^−^ T cells. Similar results were obtained in the samples from colorectal, cervical and ovarian carcinoma patients, including the expression of high levels of Foxp3 and CD25, and lower levels of CD127 on Tim-3^+^ CD4 T cells from TILs (Figure S3B and data not shown). These data strongly indicate that Tim-3 could represent a novel surface marker to define the regulatory T cell subset of CD4 T cells in human tumors.

In contrast, the majority of Tim-3^+^ CD4 T cells isolated from the peripheral blood of healthy donors and cancer patients did not express the Treg-related molecules CD25 and Foxp3. Although a trend toward increased levels of these Treg-related molecules was observed on Tim-3^+^ CD4 T cells in NILs, their frequency and intensity were markedly lower than the levels observed on the corresponding TILs (Figure 3 and S5). The differential phenotypic features of Tim-3-expressing cells in blood, NILs and TILs suggest that upregulation of Tim-3 on CD4 T cells may occur in the tumor environment, and in turn, the regulatory properties of these cells may contribute to the immunosuppressive environment in tumors.

A recent study using mouse skin transplantation model showed that the presence of Tim-3^+^PD-1^+^ Tregs *in situ* were crucial for induction of tolerance and restraining allograft rejection [14]. To determine whether Tim-3^+^PD-1^+^ Tregs exist in human tumor tissues, we compared the expression of Foxp3 in Tim-3 single positive (Tim-3 SP), PD-1 single positive (PD-1 SP), Tim-3 and PD-1 double positive cells (DP) and Tim-3 and PD-1 double negative (DN) cells. Interestingly, Tim-3 and PD-1 DP cells isolated from both NILs and TILs expressed Tim-3 at comparable levels to Tim-3 SP cells, while the PD-1 SP as well as Tim-3 and PD-1 DN cells expressed negligible levels of Foxp3 (Figure S6). Therefore, Tim-3, but not PD-1, marks the population of Foxp3^+^ T cells in the tumor microenvironment. On the other hand, PD-1^+^Tim-3^−^ cells may represent the *bona fide* population of exhausted CD4 T cells in tumor tissue.

Another recent study showed that Tim-3^+^ TILs expressed negligible levels of Foxp3 [16]; the discrepancy between this previous report and the results of this study may be due to differences in the clinical stages of the patients and the anatomic regions of the specimens analyzed. Therefore, we examined the distribution of Tim-3^+^ CD4 cells throughout the tumor tissues using multi-color immunofluorescence, paying particular attention to their micro-anatomic location. The majority of Tim-3^+^ CD4 T cells in the peritumoral stroma did not express Foxp3, whereas most Tim-3^+^ CD4 T cells in the cancer nest co-stained brightly with Foxp3 (Figure 3C). The accumulation of Tim-3^+^Foxp3^+^ CD4 T cells in the cancer nest other than in peritumoral stroma implied that Tim-3^+^ Tregs could be induced during tumor progression. In support of this hypothesis, we found that the percentage of Foxp3^+^/Tim-3^+^ CD4 T cells (Foxp3^+^/Tim-3^+^%) in TILs correlated positively with the TNM stage of the HCC patients. The 18 patients for whom Tim-3 and Foxp3 data were available were divided into two groups, according to the median Foxp3^+^/Tim-3^+^% value in TILs. In the group with a low Foxp3^+^/Tim-3^+^%, 8 out of 9 patients had an early TNM stage. In contrast, 7 out of the 9 patients from the high Foxp3^+^/Tim-3^+^% group belonged to the advanced TNM stages group (*P* = 0.015, Table S5). Similar trends were also observed using other parameters of Tim-3^+^ Tregs as the comparison index, albeit the *P* values for these analyses did not reach statistical significance (Table S5).

### CD4^+^Tim-3^+^ Cells Isolated from TILs Exhibit Suppressive Activity

**Figure 4:**
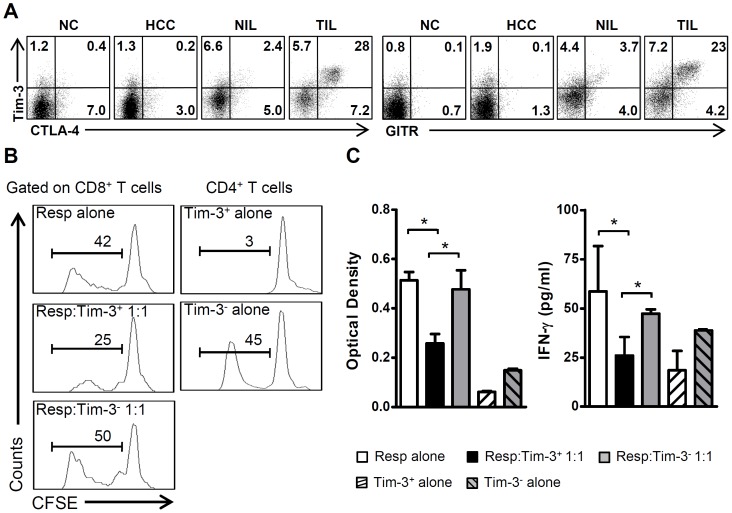
CD4^+^Tim-3^+^ T cells isolated from TILs exhibit suppressive activity. A . Representative FACS analysis showing that most tumor-infiltrating Tim-3^+^ CD4 T cells expressed the Treg functional markers CTLA-4 (left) and GITR (right), *n* = 6. **B**. CD4^+^Tim-3^+^ T cells suppressed autologus tumor-infiltrating CD8^+^ T cells. CD4^+^Tim-3^+^, CD4^+^Tim-3^−^ and CD8^+^ T cells were sorted from TILs, and the suppression assay was performed as described in Materials and Methods. The ratios of responder to suppressor cells were indicated. Cell proliferation was tracked by monitoring the decrease in CFSE fluorescence, *n* = 4. The numbers in the histograms indicate the percentage of proliferating cells. **C**. The BrdU incorporation assay supported the suppressive function of CD4^+^Tim-3^+^ T cells isolated from TILs. IFN-γ production in the indicated culture supernatants was quantified by ELISA, *n* = 4. Bars indicate the SEM; *, *P*<0.05.

To determine whether tumor-derived Tim-3^+^ CD4 T cells are functional Tregs, we first examined the expression of functional inhibitory markers of Tregs on these cells [30], [38]. Tim-3^+^ CD4 T cells from TILs expressed high levels of Cytotoxic T lymphocyte antigen-4 (CTLA-4) and glucocorticoid-induced TNF-related receptor (GITR) whereas Tim-3^+^ CD4 T cells from NILs did not express high levels of these inhibitory markers (Figure 4A), implying that tumor-derived Tim-3^+^ CD4 T cells are functional Tregs. To confirm the inhibitory activity of Tim-3^+^ Tregs, we examined their ability to suppress the proliferation and IFN-γ production of autologous tumor-infiltrating CD8^+^ T cells. Tumor-derived CD4 T cells were sorted into Tim-3^+^ and Tim-3^−^ subsets, and then cocultured with responder cells on anti-CD3/CD28 stimulation for 5 days. The CFSE assay showed that tumor-derived Tim-3^+^CD4^+^ cells inhibited the proliferation of CD8^+^ T cells, whereas Tim-3^−^CD4 T cells had no effect on the proliferation of CD8^+^ T cells (Figure 4B). In contrast to the robust proliferation of Tim-3^−^ counterparts, tumor-derived Tim-3^+^CD4 T cells were anergic to anti-CD3/CD28 stimulation, characteristics shared by “classical” human Treg cells [40]. Similar results were obtained in complementary experiments using the BrdU incorporation assay (Figure 4C). Furthermore, we observed that tumor-derived Tim-3^+^CD4^+^ cells, but not their Tim-3^−^ counterparts, suppressed production of IFN-γ by T cells (Figure 4C). Thus, Tim-3 can be used as a biomarker to identify functional Treg cells in human tumor tissues.

Notably, we observed that Tim-3^+^ CD4 T cells isolated from peripheral blood did not suppress the proliferation and IFN-γ production of T cells (data not shown), which is in line with our data indicating that circulating Tim-3^+^ CD4 T cells exhibit different functional and phenotypic features to tumor-derived Tim-3^+^ cells (Figure 2, 3 and 4A).

To further compared between Tim-3^+^ and Tim-3^−^ Foxp3^+^ CD4 T cells, we examined the expression of CTLA-4, GITR and PD-1 in these two populations. The results showed that Tim-3^+^Foxp3^+^ CD4 T cells expressed higher levels of CTLA-4, GITR and PD-1 in comparison with Tim-3^−^Foxp3^+^ CD4 T cells (Figure S7). These data suggest that Tim-3^+^Foxp3^+^ CD4 T cells in tumor tissue might represent a group of Tregs different from the Tim-3^−^Foxp3^+^ CD4 T cells.

### Close Interaction between Tim-3^+^CD4^+^ Cells and Galectin-9^+^ Cells in HCC

**Figure 5:**
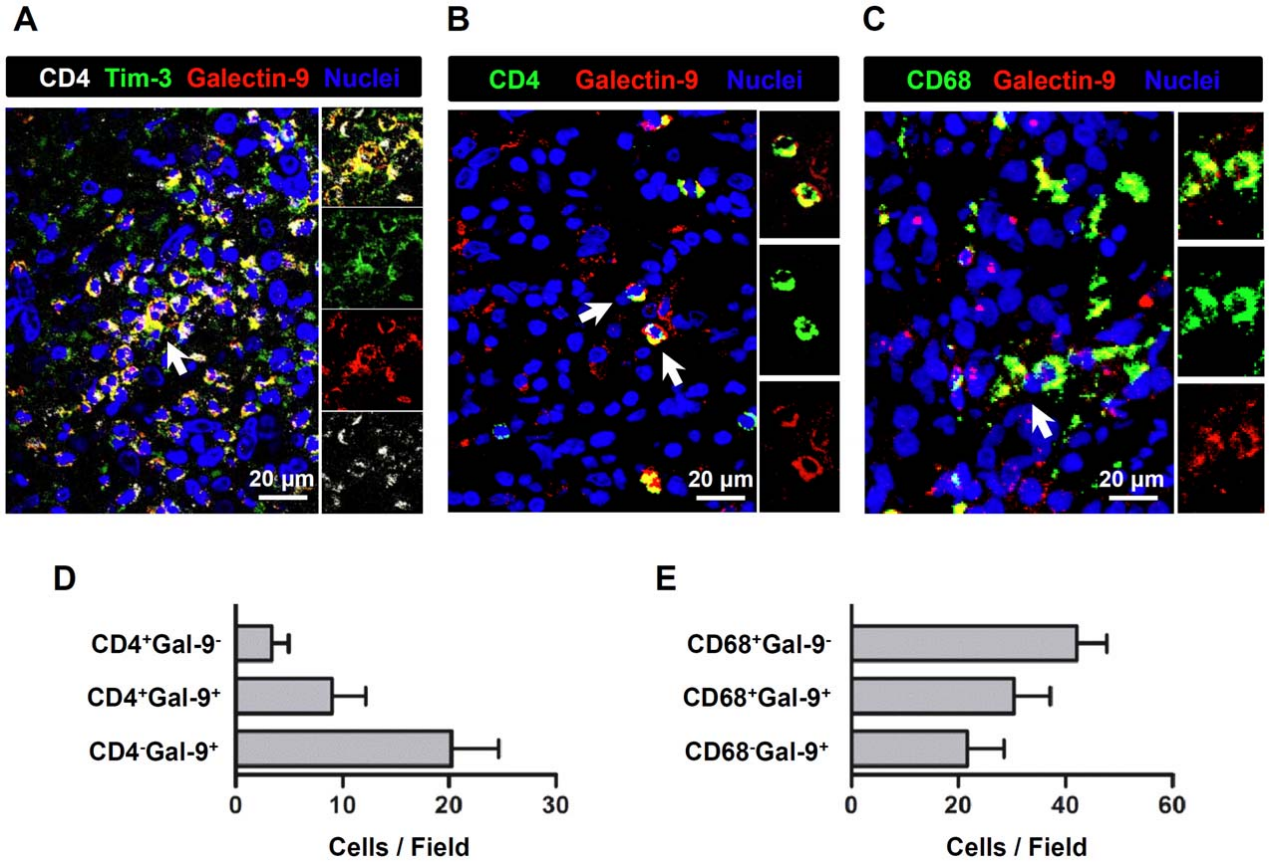
Close interaction between Tim-3^+^CD4^+^ cells and galectin-9^+^ cells in HCC tumor tissue. A . Paraffin-embedded HCC tumor sections were subjected to multi-color immunofluorescence staining for CD4 (white), Tim-3 (green), galectin-9 (red) and nuclei (DAPI, blue). Confocal analysis showed that Tim-3^+^CD4^+^ cells were in close contact with galectin-9^+^ cells in the tumor nest. **B**. Paraffin-embedded HCC tumor sections were subjected to multi-color immunofluorescence staining for CD4 (green), galectin-9 (red) and nuclei (DAPI, blue). Confocal analysis indicated that galectin-9 was expressed by the tumor-infiltrating CD4^+^ cells. **C**. Paraffin-embedded HCC tumor sections were subjected to multi-color immunofluorescence staining for CD68 (green), galectin-9 (red) and nuclei (DAPI, blue). Confocal analysis indicated that galectin-9 was expressed by tumor-infiltrating CD68^+^ macrophages. White arrows in A–C indicate the magnified cells. Images are representative of 13 samples. For the quantification of CD4^+/−^galectin-9^+/−^ cells **(D)** or CD68^+/−^galectin-9^+/−^ **(E)** cells, eight CD4 or CD68 hot-spot fields per slide were taken with 40× oil-immersed objective lens. Numbers of each subset cells were counted manually by two independent, blinded observers. Gal-9 in D and E is short for galectin-9.

The Tim-3 ligand, galectin-9, has been linked with tumor progression [41]. To explore the potential effect of galectin-9 on Tim-3^+^ cells in tumors *in situ*, we examined the distribution of galectin-9 and Tim-3 in HCC tumor tissues using multi-color immunofluorescence and confocal microscopy. Interestingly, we found that many immune cells infiltrating the tumors coexpressed Tim-3 and galectin-9 (Figure 5A), implying that Tim-3 and galectin-9 may interact with each other in an autocrine manner. Both the tumor-infiltrating CD4^+^ cells and macrophages expressed abundant levels of galectin-9 (Figure 5B–5E). Typically, a direct interaction between the Tim-3^+^CD4^+^ cells and galcetin-9^+^ cells was observed (Figure 5A), indicating that Tim-3 interacts with its ligand galectin-9 *in vivo*.

## Discussion

Tim-3 has recently been identified as an important player in the CD8 T cell exhaustion that occurs in chronic disease states such as chronic viral infection and cancer [13], [17], [19]. The present study showed that the expression of Tim-3 is upregulated on tumor-infiltrating CD4 T cells. These Tim-3^+^ CD4 T cells produced lower levels of Th1-type cytokines whereas expressed higher levels of CD25, Foxp3, CTLA-4 and GITR, compared with Tim-3^−^ CD4 T cells. Moreover, Tim-3^+^ CD4 T cells significantly suppressed autologous CD8 T cells. Interestingly, Tim-3^+^Foxp3^+^ CD4 T cells were preferentially distributed in the tumor nest, rather than the peritumoral stroma. These findings indicate that Tim-3 expression on tumor-derived CD4 T cells defines regulatory T cells which can contribute to the immunosuppressive tumor micromilieu.

Immunosuppression mediated by Tregs is a key facilitator of tumor immune evasion [30], [42], [43]; however, knowledge of the phenotypic characteristics of tumor-associated Tregs in human tumors remains limited [44], [45]. In the current study, we demonstrated that Tim-3 can serve as a novel surface marker to define tumor-infiltrating Tregs. Tim-3^+^ CD4 TILs produced lower levels of IFN-γ and IL-2, relative to their Tim-3^−^ counterparts. In contrast, over 60% of tumor-infiltrating Tim-3^+^ CD4 T cells expressed CD25, and ∼80% of Tim-3^+^ CD4 TILs were CD127^low^, two characteristics shared by human Treg cells [30], [36]–[38]. Most of the Tim-3^+^ CD4 TILs expressed the Treg-specific transcription factor Foxp3, and exhibited suppressive activity. Unlike CD25 and CD127, which characterize Treg in both peripheral blood and tumor tissues, Tim-3 is not expressed by circulating Foxp3^+^ CD4 T cells, indicating that Tim-3 is specifically expressed on tumor associated Tregs. In addition, tumor-infiltrating Tim-3^+^Foxp3^+^ CD4 T cells expressed higher levels of CTLA-4, GITR and PD-1 compared with Tim-3^−^Foxp3^+^ CD4 T cells. It has been reported that Tim-3 expression on Tregs was upregulated upon TCR activation [18]. Activated tumor-infiltrating Tregs were found to express higher levels of CTLA-4, GITR and PD-1 [46]–[48], which were observed in the Tim-3^+^Foxp3^+^ CD4 T cells. Therefore, it’s possible that Tim-3^+^ Foxp3^+^ CD4 T cells in tumor tissue might represent the highly activated Tregs that possess potent inhibitory activity. In support of our results, a recent study illustrated the accumulation of Tim-3^+^PD-1^+^ Tregs in the tumor tissues of lung cancer patients [18]. Similar to these findings, Tim-3^+^Foxp3^+^ Tregs have also been observed under other circumstances characterized by persistent antigen stimulation, such as during chronic viral infection and allograft rejection [14], [15]. Together, these studies imply that Tim-3-expressing Tregs may be involved in a diverse range of human diseases. The Tim-3 ligand galectin-9 has been found to promote the expansion of regulatory T cells; however, the exact mechanism by which galectin-9 regulates Tregs is not clear [13], [15], [49], [50]. In this study, we found that Tim-3 is specifically expressed on tumor-associated Tregs, which may render these cells susceptible to regulation by galectin-9. In support of this hypothesis, we observed that Tim-3^+^ cells were in close contact with galectin-9^+^ immune cells in tumors *in vivo*. The galectin-9 expression level correlated positively with the Foxp3 expression level in tumors *in situ* (data not shown). Treatment of T cells with galectin-9 augmented Foxp3 expression *in vitro*, as previously reported (data not shown). Hence, galectin-9 may promote Treg expansion through the Tim-3-galectin-9 interaction. In contrast, galectin-9 eliminates Tim-3^+^ effector T cells (Teffs) by triggering their apoptosis [7], [51], [52]. In this case, the Treg-to-Teff ratio would increase, coinciding with our finding that a higher Foxp3/Tim-3 ratio was associated with less favorable clinical parameters in HCC patients. Thus, the Tim-3-galectin-9 pathway could regulate homeostasis of the immune response by balancing the Treg and Teff responses in the tumor microenvironment.

The specific nature of the tumor micromilieu can profoundly determine the phenotype and function of immune cells in tumor pathology [26], [27], [53], [54]. Our previous studies showed that activated monocytes in the peritumoral stroma can promote the expansion of proinflammatory Th17 cells [27]; whereas macrophages in the tumor nest induce immunosuppressive Tregs [28]. Paying particular attention to the micro-anatomic location in tumor tissues, here we observed that the majority of Tim-3^+^CD4^+^ cells in the peritumoral stroma were Foxp3^−^, whereas the majority of Tim-3^+^CD4^+^ cells in the cancer nest co-expressed Foxp3. Furthermore, fewer Tim-3^+^Foxp3^+^ CD4 T cells were observed in the tumors of early TNM stage HCC patients, consistent with another report of negligible Tim-3 expression by Foxp3^+^ TILs isolated from relatively early-stage HCC patients [16]. We propose that Tim-3^+^ CD4 T cells exhibit different phenotypes and functions during the process of tumor progression. Early activated Tim-3^+^ Teffs in the peritumoral stroma can secrete effector cytokines such as IFN-γ, which may exert an antitumor effect in this area [53], [55]. However, IFN-γ-induced expression of galectin-9 in tumor-associated macrophages [16] promotes Tim-3^+^ Treg expansion, which in turn eliminates and dampens the effects of Teffs in the tumor nest. Therefore, different tissue contexts may determine whether Tim-3^+^ CD4 T cells exert a beneficial or detrimental effect on tumor immunopathogenesis.

Emerging Tim-3 function in tumor immunity is being discovered [7], [51], [52]. Treatment with anti-Tim-3 Ab alone or in combination with anti-CTLA-4 and/or PD-1 Abs significantly suppressed tumor growth in mice [17], [19], [21], [56]. In addition to the inhibitory role of Tim-3 in the immune response, Tim-3 has also been found to exert an antitumor immune response [22]–[24]. The results of the present study reveal that different tissue contexts may determine the phenotype and function of Tim-3^+^ CD4 T cells in the tumor microenvironment. Fine-tuning the Tim-3 pathway could potentially help to develop a novel antitumor therapy, to simultaneously restore the functions of Teffs and restrain the immunosuppressive effects of Tregs.

## Supporting Information

Figure S1 **Isotype control for Tim-3 expression.** Isotype controls for Tim-3 were shown for CD4 T cells isolated from PBMCs from normal controls (NC), PBMCs from HCC patients (HCC), nontumor-infiltrating lymphocytes (NIL) and tumor-infiltrating lymphocytes (TILs). Images are representative of at least 31 samples in each group.
(TIF)

Figure S2 **Proportion of Tim-3^+^ lymphocyte subsets in Tim-3^+^ cells.** Frequency of T cells (CD3^+^), CD4 T cells (CD3^+^CD4^+^), CD8 T cells (CD3^+^CD8^+^), NKT cells (CD3^+^CD56^+^) and NK cells (CD3^−^CD56^+^) in Tim-3^+^ lymphocytes isolated from PBMCs from normal controls (NC), PBMCs from HCC patients (HCC), nontumor-infiltrating lymphocytes (NIL) and tumor-infiltrating lymphocytes (TILs). At least 12 samples were tested in each group. Bars indicate the SEM. *, *P*<0.05, ***, *P*<0.001.
(TIF)

Figure S3 **Tim-3 is upregulated on tumor-infiltrating Foxp3^+^ CD4 T cells isolated from colon carcinoma, cervical cancer and ovarian cancer patients. A.** Proportion of Tim-3^+^ CD4 T cells isolated from paired non-tumor-infiltrating lymphocytes (NILs) and tumor-infiltrating lymphocytes (TILs). Each line represents a single case; *, *P*<0.05. **B.** Representative FACS analysis of Tim-3 and Foxp3 expression in NILs and TILs isolated from patients with colon carcinoma (*n* = 4), cervical cancer (*n* = 3) and ovarian cancer (*n* = 3).
(TIF)

Figure S4 **Proportion of Th2, Th17, Th1 and Treg cells in Tim-3** ^−^ **and Tim-3^+^ CD4 T cells.** Statistical analysis showed the percentage of IL-4^+^, IL-17^+^, IFN-γ^+^, IL-2^+^ and Foxp3^+^ cells in Tim-3^−^ and Tim-3^+^ CD4 T cells from non-tumor-infiltrating lymphocytes (NIL) and tumor-infiltrating lymphocytes (TIL) isolated from HCC patients. At least 6 samples were tested in each group. Bars indicate the SEM; **, *P*<0.01; ***, *P*<0.001.
(TIF)

Figure S5 **Statistical analysis showed the mean percentage of CD25^+^ cells (A) and CD127^low^ cells (B) in the Tim-3 positive or Tim-3 negative CD4 T cell subsets (left), and the proportion of Tim-3^+^ cells in the CD25 (A) and CD127 (B) high or negative/low CD4 T cell subsets (right) isolated from HCC patients. At least 12 samples were tested in each group.** Bars indicate the SEM. *, *P*<0.05; **, *P*<0.01; ***, *P*<0.001.
(TIF)

Figure S6 **Frequency of Foxp3 expression in Tim-3 single positive (Tim-3 SP), Tim-3 and PD-1 double positive (DP), PD-1 single positive (PD-1 SP), and Tim-3 and PD-1 double negative (DN) CD4 T cell subsets from non-tumor-infiltrating lymphocytes (NIL,** ***n*** **= 4) and tumor-infiltrating lymphocytes (TIL,** ***n*** **= 4) isolated from HCC patients.** Bars indicate the SEM; *, *P*<0.05; **, *P*<0.01; ***, *P*<0.001.
(TIF)

Figure S7 **Expression of CTLA-4, GITR and PD-1 in Tim-3^+^Foxp3^+^ and Tim-3** ^−^ **Foxp3^+^ CD4 T cells.** Tumor-infiltrating lymphocytes were first gated on CD4^+^Foxp3^+^ T cells. Expression of CTLA-4, GITR and PD-1 were compared between Tim-3^+^Foxp3^+^ and Tim-3^−^Foxp3^+^ CD4 T cells. FACS images are representative of 4 samples. Bars indicate the SEM; *, *P*<0.05; **, *P*<0.01.
(TIF)

Table S1 **Clinical characteristics of the four colon cancer patients.**
(DOC)

Table S2 **Clinical characteristics of the three cervical cancer patients.**
(DOC)

Table S3 **Clinical characteristics of the three ovarian cancer patients.**
(DOC)

Table S4 **Fluorochrome-conjugated antibodies used in flow cytometry.**
(DOC)

Table S5 **Correlation between Tim-3^+^ Tregs and TNM stages in HCC.**
(DOC)
