# Supplementary figures and images for: Tim-3 Expression Defines Regulatory T Cells in Human Tumors
Source: PLoS One. 2013 Mar 5;8(3):e58006. doi: 10.1371/journal.pone.0058006 (PMC3589491; doi:10.1371/journal.pone.0058006)

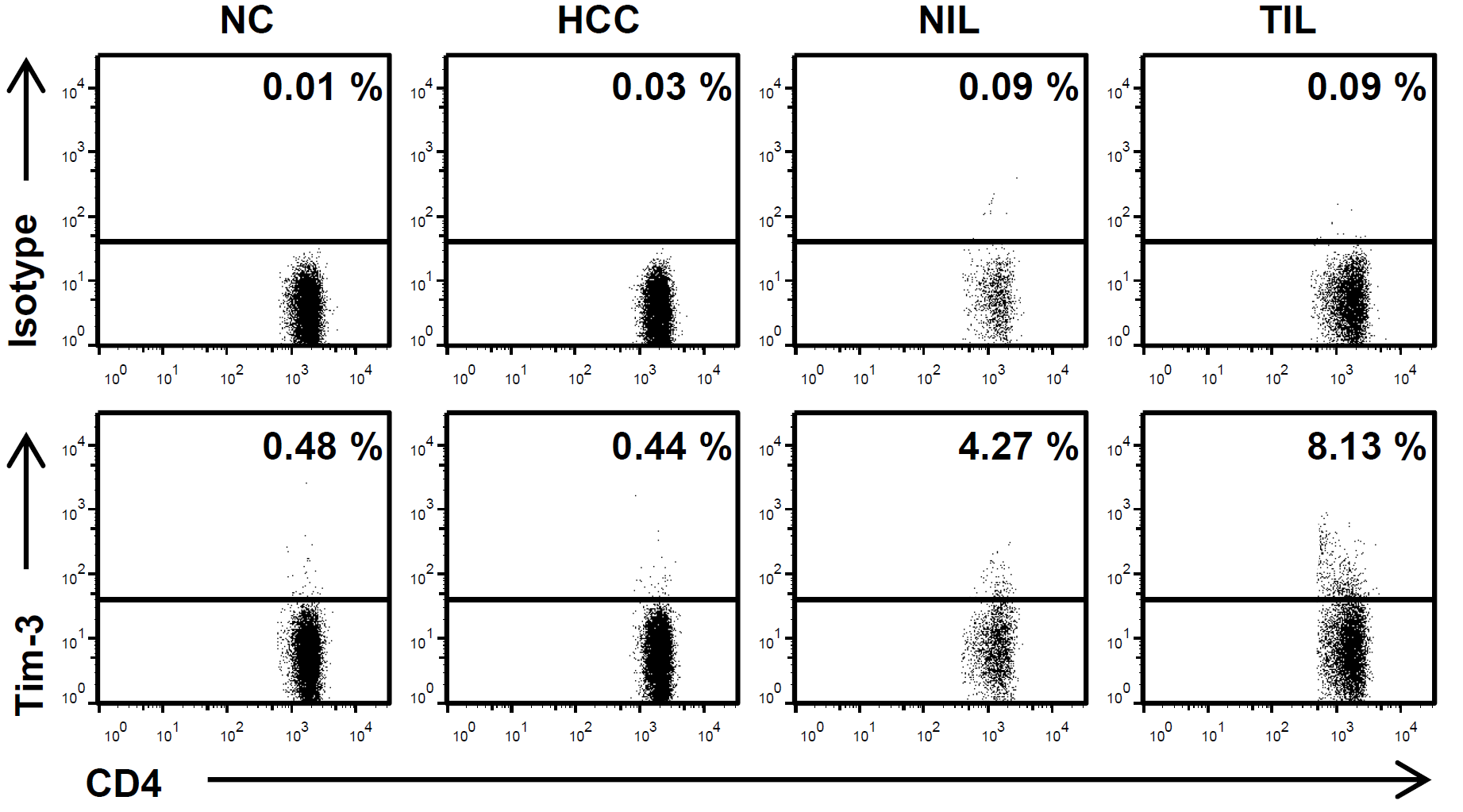

Supplement: Figure S1 — Isotype control for Tim-3 expression. Isotype controls for Tim-3 were shown for CD4 T cells isolated from PBMCs from normal controls (NC), PBMCs from HCC patients (HCC), nontumor-infiltrating lymphocytes (NIL) and tumor-infiltrating lymphocytes (TILs). Images are representative of at least 31 samples in each group. (TIF) [file pone.0058006.s001.tif]

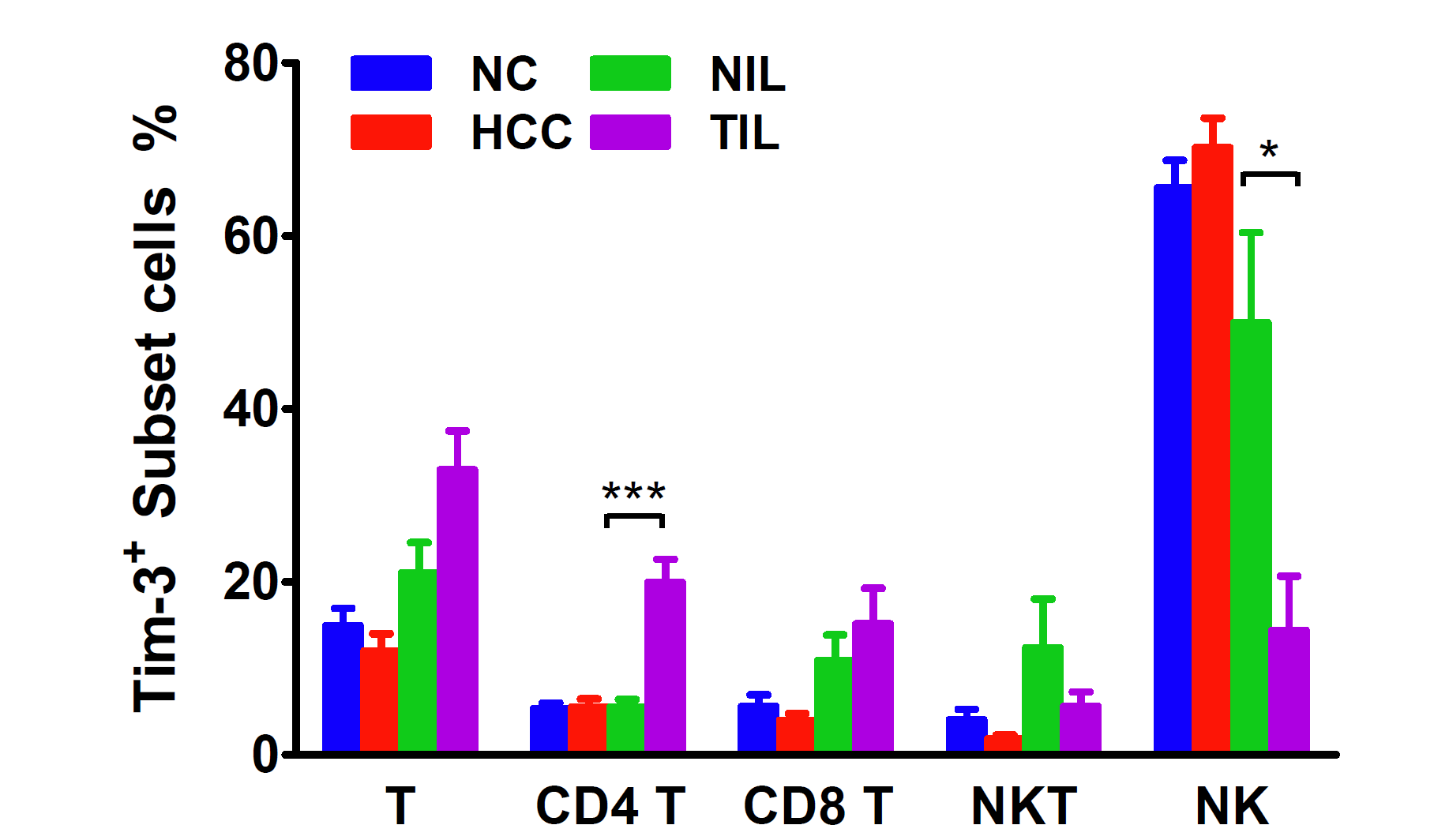

Supplement: Figure S2 — Proportion of Tim-3+ lymphocyte subsets in Tim-3+ cells. Frequency of T cells (CD3+), CD4 T cells (CD3+CD4+), CD8 T cells (CD3+CD8+), NKT cells (CD3+CD56+) and NK cells (CD3−CD56+) in Tim-3+ lymphocytes isolated from PBMCs from normal controls (NC), PBMCs from HCC patients (HCC), nontumor-infiltrating lymphocytes (NIL) and tumor-infiltrating lymphocytes (TILs). At least 12 samples were tested in each group. Bars indicate the SEM. *, P<0.05, ***, P<0.001. (TIF) [file pone.0058006.s002.tif]

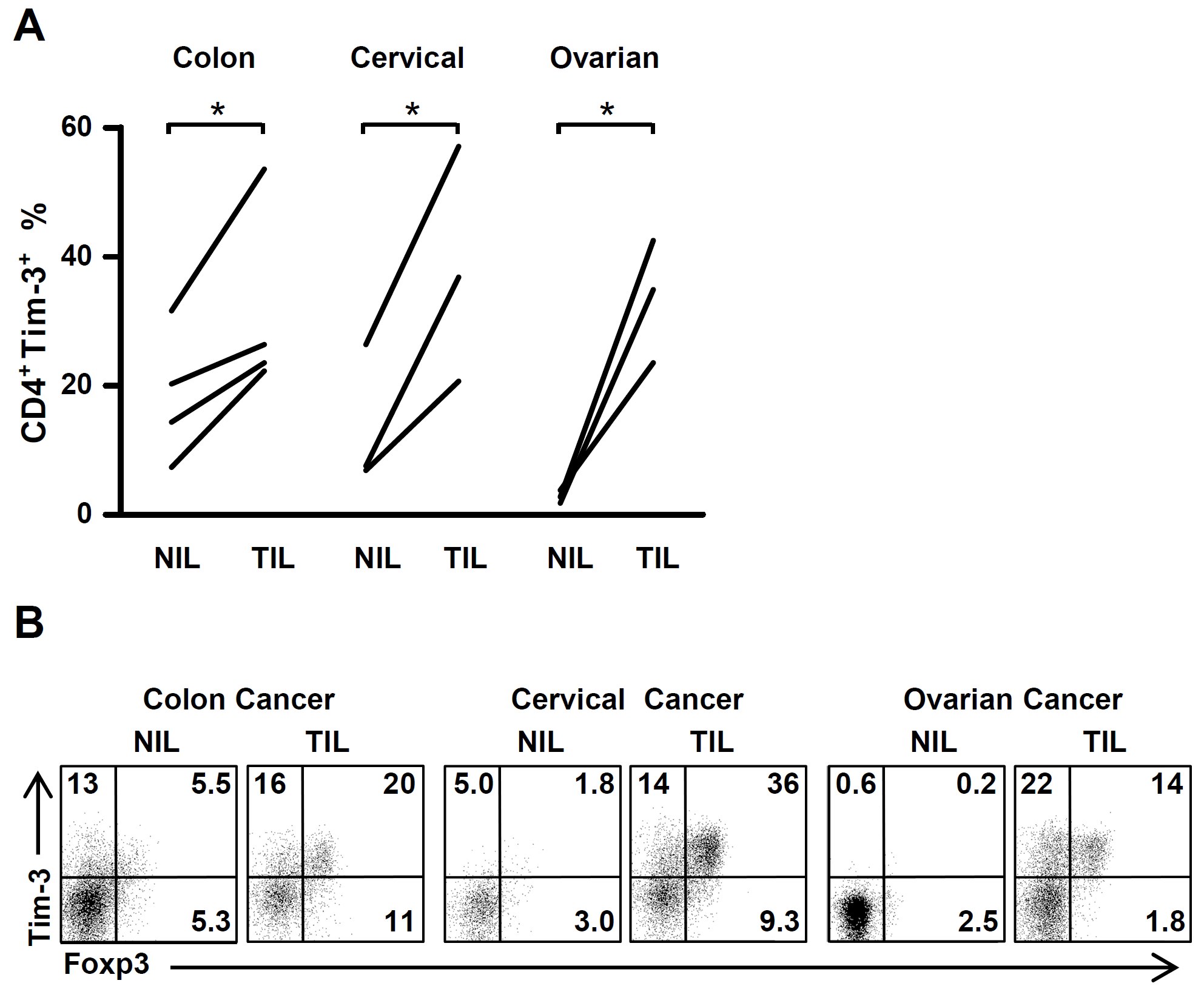

Supplement: Figure S3 — Tim-3 is upregulated on tumor-infiltrating Foxp3+ CD4 T cells isolated from colon carcinoma, cervical cancer and ovarian cancer patients. A. Proportion of Tim-3+ CD4 T cells isolated from paired non-tumor-infiltrating lymphocytes (NILs) and tumor-infiltrating lymphocytes (TILs). Each line represents a single case; *, P<0.05. B. Representative FACS analysis of Tim-3 and Foxp3 expression in NILs and TILs isolated from patients with colon carcinoma (n = 4), cervical cancer (n = 3) and ovarian cancer (n = 3). (TIF) [file pone.0058006.s003.tif]

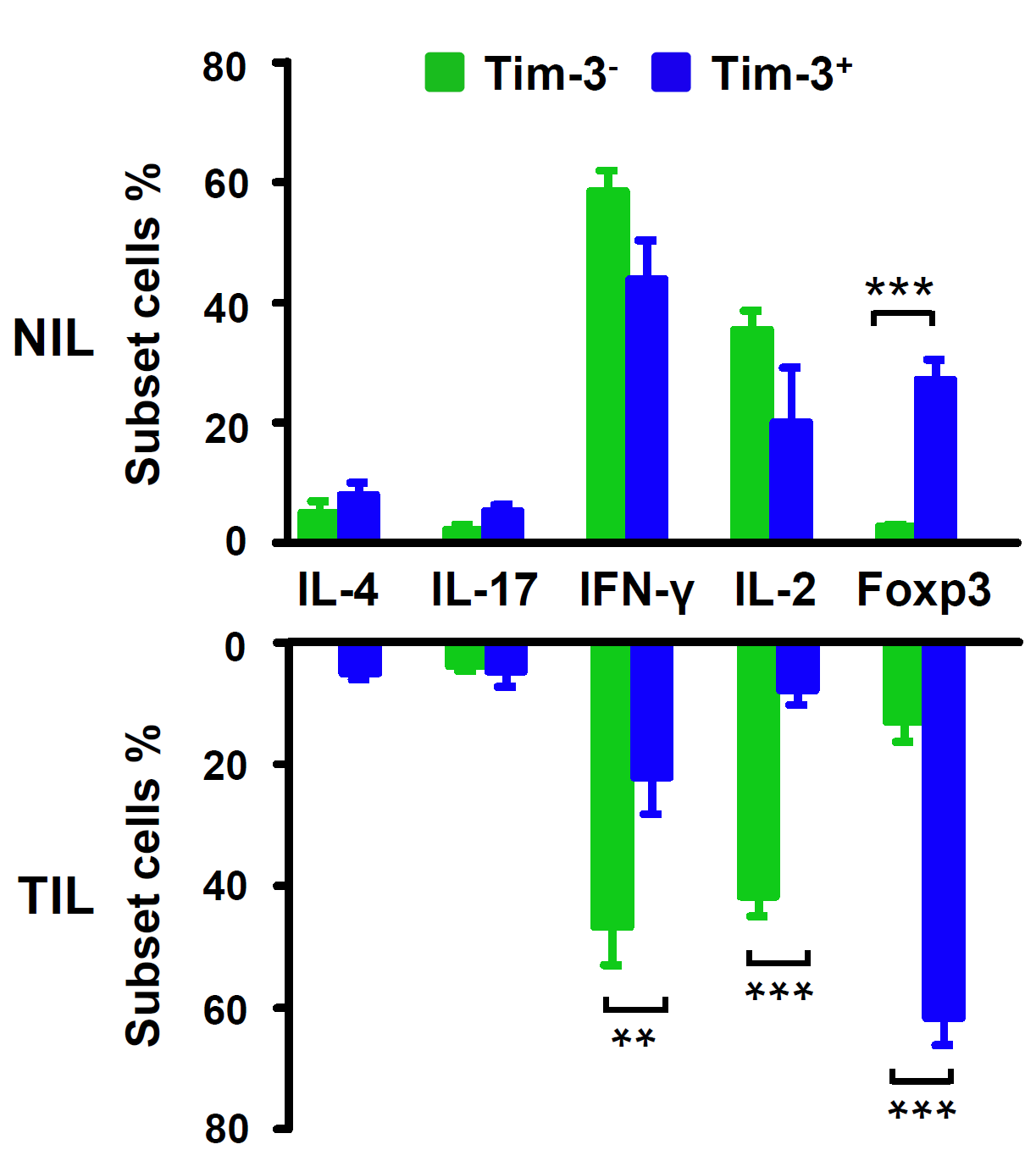

Supplement: Figure S4 — Proportion of Th2, Th17, Th1 and Treg cells in Tim-3 − and Tim-3+ CD4 T cells. Statistical analysis showed the percentage of IL-4+, IL-17+, IFN-γ+, IL-2+ and Foxp3+ cells in Tim-3− and Tim-3+ CD4 T cells from non-tumor-infiltrating lymphocytes (NIL) and tumor-infiltrating lymphocytes (TIL) isolated from HCC patients. At least 6 samples were tested in each group. Bars indicate the SEM; **, P<0.01; ***, P<0.001. (TIF) [file pone.0058006.s004.tif]

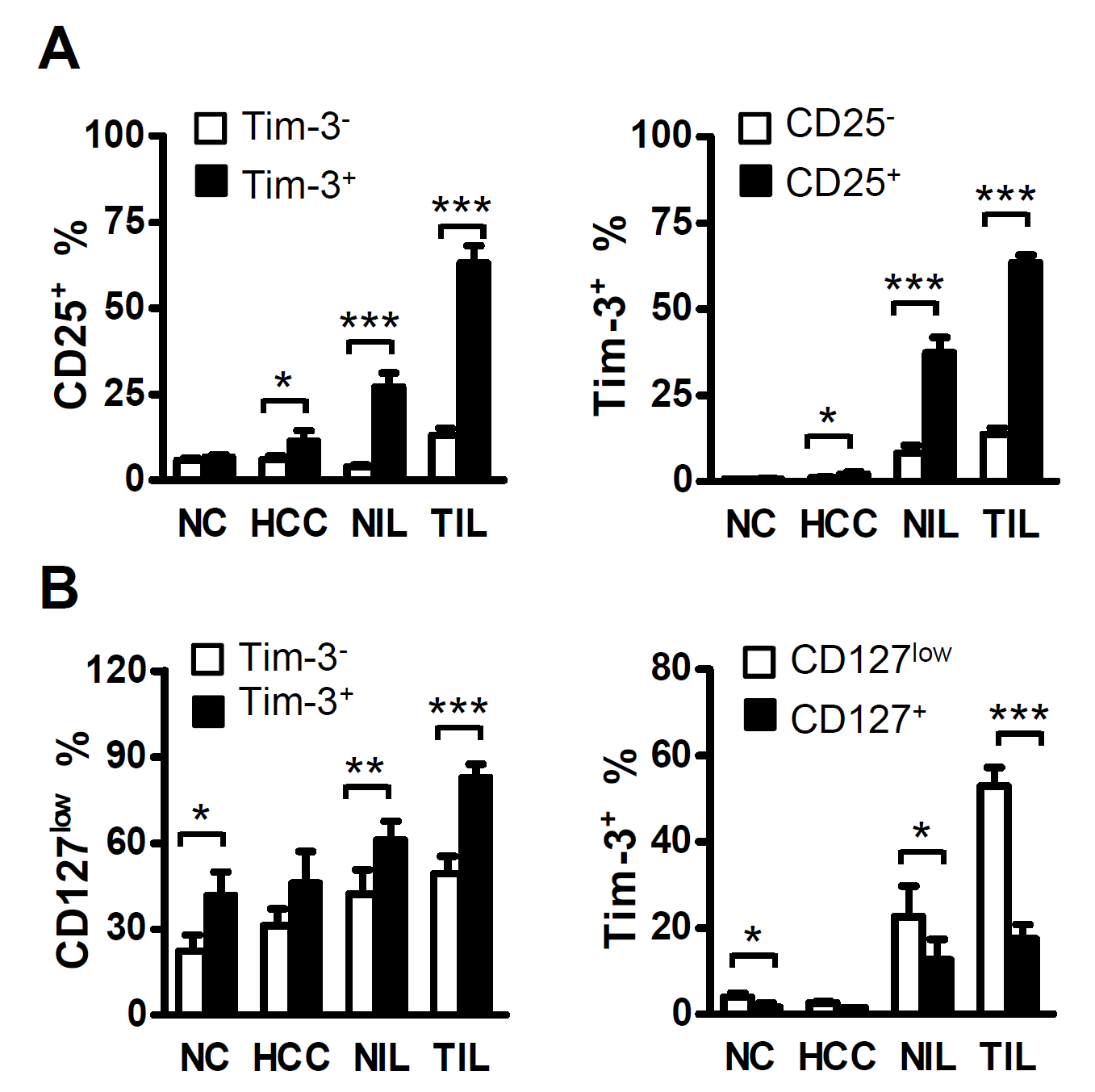

Supplement: Figure S5 — Statistical analysis showed the mean percentage of CD25+ cells (A) and CD127low cells (B) in the Tim-3 positive or Tim-3 negative CD4 T cell subsets (left), and the proportion of Tim-3+ cells in the CD25 (A) and CD127 (B) high or negative/low CD4 T cell subsets (right) isolated from HCC patients. At least 12 samples were tested in each group. Bars indicate the SEM. *, P<0.05; **, P<0.01; ***, P<0.001. (TIF) [file pone.0058006.s005.tif]

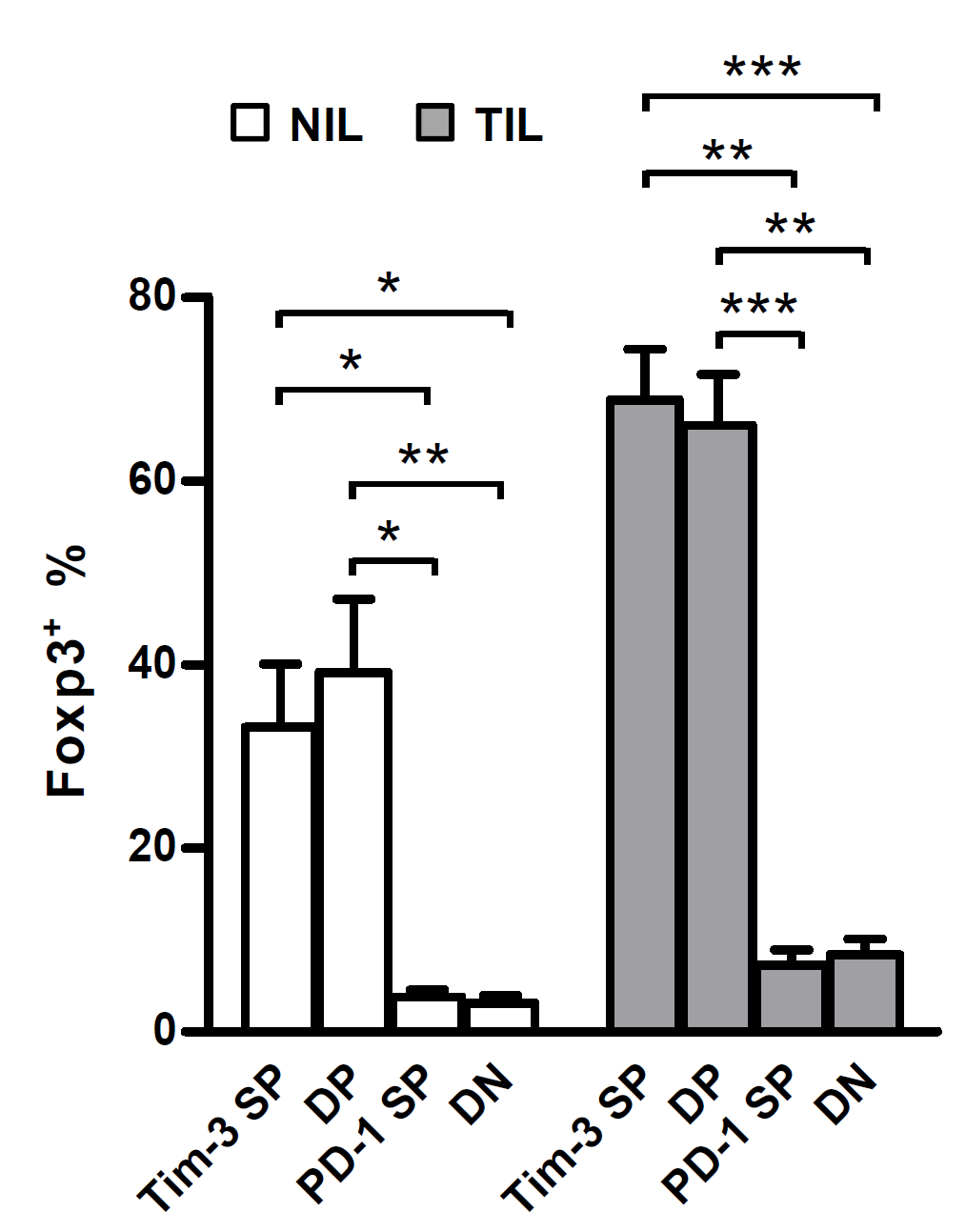

Supplement: Figure S6 — Frequency of Foxp3 expression in Tim-3 single positive (Tim-3 SP), Tim-3 and PD-1 double positive (DP), PD-1 single positive (PD-1 SP), and Tim-3 and PD-1 double negative (DN) CD4 T cell subsets from non-tumor-infiltrating lymphocytes (NIL, n = 4) and tumor-infiltrating lymphocytes (TIL, n = 4) isolated from HCC patients. Bars indicate the SEM; *, P<0.05; **, P<0.01; ***, P<0.001. (TIF) [file pone.0058006.s006.tif]

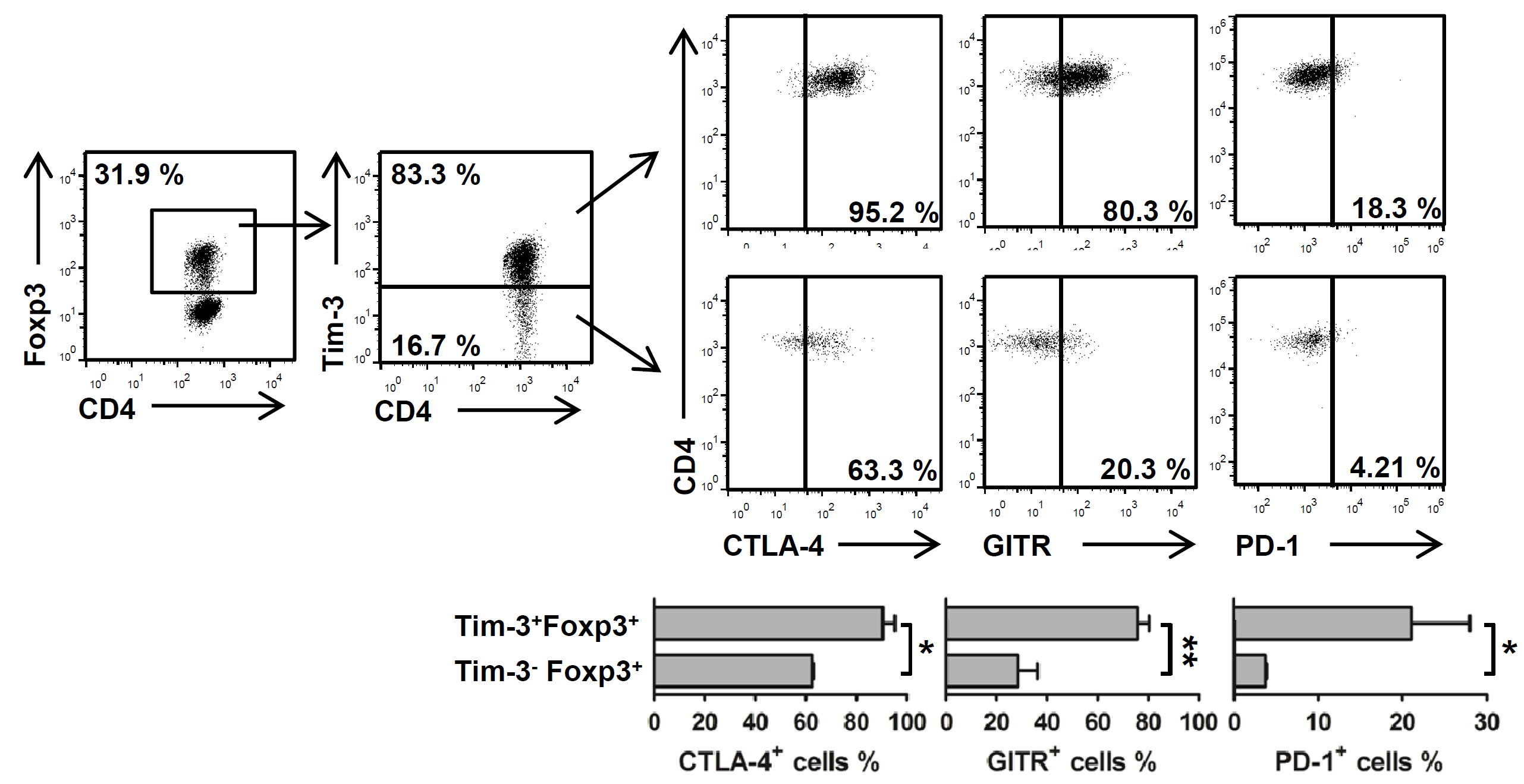

Supplement: Figure S7 — Expression of CTLA-4, GITR and PD-1 in Tim-3+Foxp3+ and Tim-3 − Foxp3+ CD4 T cells. Tumor-infiltrating lymphocytes were first gated on CD4+Foxp3+ T cells. Expression of CTLA-4, GITR and PD-1 were compared between Tim-3+Foxp3+ and Tim-3−Foxp3+ CD4 T cells. FACS images are representative of 4 samples. Bars indicate the SEM; *, P<0.05; **, P<0.01. (TIF) [file pone.0058006.s007.tif]
