# Supplementary material for: Tim-3 Expression Defines Regulatory T Cells in Human Tumors
Source: PLoS One. 2013 Mar 5;8(3):e58006. doi: 10.1371/journal.pone.0058006 (PMC3589491; doi:10.1371/journal.pone.0058006)
Supplement: Table S1 — Clinical characteristics of the four colon cancer patients. (DOC) [file pone.0058006.s008.doc]

| **Table S1. Clinical characteristics of the four colon cancer patients** | |
| --- | --- |
| Variable | Result |
| Cases (*n*) | 4 |
| Age, years (median, range) | 57, 41-74 |
| Gender (male/female) | 2/2 |
| Tumor (T) stage (Tis/T1/T2/T3/T4) | 0/0/0/1/3 |
| Nodal (N) status (negative/positive/Nx) | 0/4/0 |
| Distant metastases (M) (not detected/present) | 1/3 |
| Location (right side/transverse/left side/sigmoid/rectum) | 3/1/0/0/0 |
| Differentiation (well/moderate/poor) | 0/1/3 |
