# Supplementary material for: Tim-3 Expression Defines Regulatory T Cells in Human Tumors
Source: PLoS One. 2013 Mar 5;8(3):e58006. doi: 10.1371/journal.pone.0058006 (PMC3589491; doi:10.1371/journal.pone.0058006)
Supplement: Table S2 — Clinical characteristics of the three cervical cancer patients. (DOC) [file pone.0058006.s009.doc]

| **Table S2. Clinical characteristics of the three cervical cancer patients** | |
| --- | --- |
| Variable | Result |
| Cases (*n*) | 3 |
| Age, years (median, range) | 44, 32-45 |
| Histological types  (squamous/adenocarcinoma/clear cell/undifferentiated) | 2/1/0/0 |
| FIGO stage (I/II/III/IV) | 2/1/0/0 |
| Histological grade (well/moderate/poor) | 0/1/2 |
| Note: FIGO, International Federation of Gynecology and Obstetrics. | |
