# Supplementary material for: Tim-3 Expression Defines Regulatory T Cells in Human Tumors
Source: PLoS One. 2013 Mar 5;8(3):e58006. doi: 10.1371/journal.pone.0058006 (PMC3589491; doi:10.1371/journal.pone.0058006)
Supplement: Table S3 — Clinical characteristics of the three ovarian cancer patients. (DOC) [file pone.0058006.s010.doc]

| **Table S3. Clinical characteristics of the three ovarian cancer patients** | | |
| --- | --- | --- |
| Variable | Result | |
| Cases (*n*) | 3 | |
| Age, years (median, range) | 55, 43-62 | |
| Histological types  (serous, mucinous or endometrimoid/clear-cell or undifferentiated) | 3/0 | |
| FIGO stage (II/III/IV) | 0/3/0 | |
| Histological grade (well/moderate/poor) | 0/1/2 | |
| Residual disease (microscopic-0.5, 0.5-2, > 2 cm) | 0/3/0 | |
| Note: FIGO, International Federation of Gynecology and Obstetrics. | |  |
