# Supplementary material for: Tim-3 Expression Defines Regulatory T Cells in Human Tumors
Source: PLoS One. 2013 Mar 5;8(3):e58006. doi: 10.1371/journal.pone.0058006 (PMC3589491; doi:10.1371/journal.pone.0058006)
Supplement: Table S4 — Fluorochrome-conjugated antibodies used in flow cytometry. (DOC) [file pone.0058006.s011.doc]

**Table S4. Fluorochrome-conjugated antibodies used in flow cytometry**

| Antibody | Supplier |
| --- | --- |
| Alexa Fluor700-conjugated anti-human CD3 | eBioscience |
| eFluor450-conjugated anti-human CD4 | eBioscience |
| PE-Cy7-conjugated anti-human CD4 | Beckman Coulter |
| PE-Cy7-conjugated anti-human CD8 | Beckman Coulter |
| PE-conjugated anti-human Tim-3 | R&D systems |
| PE-conjugated Rat IgG2a | BD Pharmingen |
| FITC-conjugated anti-human PD-1 | eBioscience |
| FITC-conjugated anti-human IFN-γ | Beckman Coulter |
| eFluor450-conjugated anti-human IFN-γ | eBioscience |
| APC-conjugated anti-human IL-2 | BD Pharmingen |
| Alexa Fluor 647-conjugated anti-human IL-17 | eBioscience |
| FITC-conjugated anti-human IL-4 | BD Pharmingen |
| FITC-conjugated anti-human CD25 | Beckman Coulter |
| PE-Cy7-conjugated anti-human CD25 | eBioscience |
| FITC-conjugated anti-human CD127 | eBioscience |
| APC-conjugated anti-human Foxp3 | eBioscience |
| FITC-conjugated anti-human Foxp3 | eBioscience |
| APC-conjugated anti-human GITR | eBioscience |
| APC-conjugated anti-human CTLA-4 | BD Pharmingen |
| Alexa Fluor 647-conjugated anti-human T-bet | eBioscience |
| Pacific Orange-conjugated anti-human CD45 | Caltag Laboratories |
| FITC-conjugated Mouse IgG1 κ Isotype Control | eBioscience |
| eFluor 450-conjugated Mouse IgG1 κ Isotype Control | eBioscience |
| Alexa Fluor 647-conjugated Mouse IgG1 κ Isotype Control | eBioscience |
| PE-Cy7-conjugated Mouse IgG1κ Isotype Control | eBioscience |
| APC-conjugated Mouse IgG1 κ Isotype Control | BD Pharmingen |

Suppliers: eBioscience (San Diego, CA); BD Pharmingen (San Diego, CA); Beckman Coulter (Fullerton, CA); R&D systems (Minneapolis, MN); Caltag Laboratories, (Carlsbad, CA).
