# Supplementary material for: Tim-3 Expression Defines Regulatory T Cells in Human Tumors
Source: PLoS One. 2013 Mar 5;8(3):e58006. doi: 10.1371/journal.pone.0058006 (PMC3589491; doi:10.1371/journal.pone.0058006)
Supplement: Table S5 — Correlation between Tim-3+ Tregs and TNM stages in HCC. (DOC) [file pone.0058006.s012.doc]

| **Table S5. Correlation between Tim-3^+^ Tregs and TNM stages in HCC** | | | | |
| --- | --- | --- | --- | --- |
| Index | Group | TNM stages | | *P* value |
|  |  | I-II | III |  |
| Foxp3^+^/Tim-3^+^CD4^+^ % | Low | 8 | 1 | 0.015^†^ |
|  | High | 2 | 7 |  |
| Tim-3^+^/Foxp3^+^CD4^+^ % | Low | 6 | 3 | 0.637 |
|  | High | 4 | 5 |  |
| Foxp3^+^Tim-3^+^CD4^+^ % | Low | 7 | 2 | 0.153 |
|  | High | 3 | 6 |  |
| Tim-3^+^CD4^+^ % | Low | 7 | 2 | 0.153 |
|  | High | 3 | 6 |  |
| Foxp3^+^CD4^+^ % | Low | 6 | 3 | 0.637 |
|  | High | 4 | 5 |  |
| ^†^ Calculated by Fisher's exact test. | | | | |
